# Health Care Expenditures for Adults With Multiple Treated Chronic Conditions: Estimates From the Medical Expenditure Panel Survey, 2009

**DOI:** 10.5888/pcd10.120172

**Published:** 2013-04-25

**Authors:** Steven R. Machlin, Anita Soni

**Affiliations:** Author Affiliation: Steven R. Machlin, Agency for Healthcare Research and Quality, Center for Financing, Access, and Cost Trends, Rockville, Maryland.

## Abstract

The objective of this article is to illustrate the usefulness of Medical Expenditure Panel Survey (MEPS) data for examining variations in medical expenditures for people with multiple chronic conditions (MCC). We analyzed 2009 MEPS data to produce estimates of treated prevalence for MCC and associated medical expenditures for adults in the US civilian noninstitutionalized population (sample = 24,870). We also identified the most common dyad and triad combinations of treated conditions. Approximately one-quarter of civilian US adults were treated for MCCs in 2009; 18.3% were treated for 2 to 3 conditions and 7% were treated for 4 or more conditions. The proportion of adults treated for MCC increased with age. White non-Hispanic adults were most likely and Hispanic and Asian adults were least likely to be treated for MCC. Health care expenditures increased as the number of chronic conditions treated increased. Regardless of age or sex, hypertension and hyperlipidemia was the most common dyad among adults treated for MCC; diabetes in conjunction with these 2 conditions was a common triad. MEPS has the capacity to produce national estimates of health care expenditures associated with MCC. MEPS data in conjunction with data from other US Department of Health and Human Services sources provide information that can inform policies addressing the complex issue of MCC.

## Introduction

Chronic conditions are broadly defined as those expected to last at least 1 year and result in limitations of self-care, independent living, and social interactions or the need for ongoing medical intervention (1,2). In 2009, the top 5 most costly medical conditions in terms of health care expenditures (heart disease, trauma-related disorders, cancer, mental disorders, and chronic obstructive pulmonary disease/asthma) (3) were primarily chronic in nature. Because of factors such as the aging of the population (4) and high obesity rates (5,6), the proportion of the population with multiple chronic conditions (MCC) is likely to rise. This trend will exacerbate public policy concerns related to prevention, treatment, and costs of care for people with MCC.

The Medical Expenditure Panel Survey (MEPS) is a federal survey that has the capacity to produce nationally representative estimates of health care expenditures associated with medical conditions for the US civilian noninstitutionalized population. Previous research has used MEPS for estimates of expenditures on chronic conditions (7-9) but has not examined variations by number of chronic conditions or presented common dyads and triads of conditions. The objective of this article is to provide descriptive estimates that illustrate the usefulness of MEPS data for examining variations in medical expenditures for people with MCC. Estimates are provided according to number of MCC and selected characteristics of the adult population aged 18 years or older; the most common dyad and triad combinations of treated conditions are also identified.

## Analysis

The MEPS Household Component (MEPS-HC) is a nationally representative survey of the US civilian noninstitutionalized population that has been conducted since 1996 (10). It provides data that can be used to produce annual estimates as well as behavioral and economic analyses of health care use, expenditures, insurance coverage, sources of payment, access to care, and health care quality. The MEPS-HC uses an overlapping panel design in which a new sample panel of households is selected each year from respondents to the prior year’s National Health Interview Survey (11). Data are collected in 5 rounds of computer-assisted personal interviews that cumulatively cover a 2-year period. Typically, 1 representative from the household responds for all family members. The 2009 MEPS-HC comprises a sample of approximately 14,000 households across 2 consecutive panels with a combined overall response rate of approximately 60%. MEPS data collection and analyses are covered under the auspices of human research protocols that have institutional review board approval (12).

The set of conditions considered to be chronic in this article was developed by the US Department of Health and Human Services (DHHS) Interagency Workgroup on MCC and the Office of the Assistant Secretary of Health (13). It consists of the following conditions: arthritis, asthma, autism spectrum disorder, cancer, cardiac arrhythmias, chronic kidney disease, chronic obstructive pulmonary disease, congestive heart failure, coronary artery disease, dementia, depression, diabetes, hepatitis, HIV infection, hyperlipidemia, hypertension, osteoporosis, schizophrenia, stroke, and substance abuse disorders. DHHS used a deliberative process to select these conditions that required not only meeting the definition of chronic but also being prevalent in the population and having the potential for public health or clinical interventions.

Estimates in this report are derived on the basis of data for adults aged 18 or older in the 2009 MEPS (N = 24,870) classified according to the number of chronic conditions for which they were treated during the year (0 to 1, 2 to 3, 4 or more). The condition data used for this analysis were derived on the basis of verbatim text responses to open-ended questions about conditions reported to be associated with health care events. These reported conditions are coded according to the *International Classification of Diseases, Ninth Revision, Clinical Modification* (ICD-9-CM). Treatment of these conditions may have been received through ambulatory visits to office or hospital outpatient settings, emergency departments, hospital inpatient stays, home health services, or prescribed medicines. Expenditures in MEPS for these types of services are defined as total payments from all sources for the care provided including payments from patients (ie, out of pocket), private insurance, Medicare, Medicaid, workers’ compensation, and other sources. Data on expenditures are derived from information collected in both the MEPS-HC and the MEPS Medical Provider Component (MEPS-MPC). In the MEPS-MPC, data are collected from a sample of medical providers identified as providing care to people in the MEPS-HC (14).

We compared participants’ demographic characteristics, health care use, and expenditures between people treated for MCC (defined as 2 or more) and those treated for only 1 or no chronic conditions (ie, people not treated for 2 or more MCC). Variations in common dyad and triad combinations of treated conditions were also examined by age and sex. Less than 6% of the sample aged 18 to 44 years was treated for dyads or triads; their data are not presented. The “treated prevalence” estimates in this report should not be misconstrued as equivalent to actual chronic condition prevalence because some people may not receive treatment in a given year for a particular chronic condition or some respondents may not be aware that they (or their family member) have the condition.

All estimates were produced by using SAS version 9.2 (SAS Institute Inc, Cary, North Carolina) and SUDAAN version 10.0.1 (RTI International, Research Triangle Park, North Carolina) and were weighted to represent 231.8 million adults in the US civilian noninstitutionalized population in 2009. Standard errors of estimates were computed by using the Taylor series method (15), which takes into account the MEPS complex survey design. Differences noted in the text are significant at .05.

## Results

Approximately 25% of civilian US noninstitutionalized adults aged 18 or older were treated for MCC in 2009. A total of 18.3% were treated for 2 to 3 conditions, and 7% were treated for 4 or more conditions (Table 1).

**Table 1:** Number of Treated Chronic Conditions^a^^,^ b by Demographic Characteristics, Medical Expenditure Panel Survey, 2009

Demographic Variable	Population (in millions)	No. of Treated Chronic Conditions, % (95% CI)		

		0 to 1	2 to 3	4 or more
**≥18 y**	231.8	74.7 (73.8–75.6)	18.3 (17.5–19.1)	7.0 (6.6–7.4)
**Age group**				
18–44 y	111.1	94.2 (93.6–94.8)	5.2 (4.7–5.7)	0.6 (0.4–0.8)
45–64 y	80.3	68.8 (67.5–70.1)	24.3 (23.1–25.5)	7.0 (6.3–7.7)
≥65 y	40.3	32.9 (30.9–34.9)	42.5 (40.4–44.6)	24.6 (23.0–26.2)
**Sex **				
Male	112.3	76.9 (75.8–78.0)	17.1 (16.2–18.0)	6.1 (5.5–6.7)
Female	119.5	72.7 (71.6–73.8)	19.5 (18.5–20.5)	7.9 (7.3–8.5)
**Sex, age (y)**				
Male, 18–44	55.7	95.2 (94.5–95.9)	4.3 (3.7–4.9)	0.5 (0.3–0.7)
Male, 45–64	39.2	69.6 (67.8–71.4)	24.5 (22.8–26.2)	5.9 (5.0–6.8)
Male, ≥65	17.5	34.6 (31.7–37.5)	41.1 (38.2–44.0)	24.4 (21.8–27.0)
Female, 18–44	55.5	93.2 (92.2–94.2)	6.1 (5.2–7.0)	0.7 (0.4–1.0)
Female, 45–64	41.2	67.9 (66.3–69.5)	24.0 (22.5–25.5)	8.1 (7.1–9.1)
Female, ≥65	22.9	31.6 (29.3–33.9)	43.7 (41.1–46.3)	24.8 (22.8–26.8)
**Race **				
Hispanic	32.0	85.6 (84.4–86.8)	10.7 (9.7–11.7)	3.7 (3.2–4.2)
White, non-Hispanic	158.0	71.5 (70.4–72.6)	20.4 (19.4–21.4)	8.1 (7.5–8.7)
Black, non-Hispanic	26.5	77.6 (76.0–79.2)	16.7 (15.3–18.1)	5.7 (4.9–6.5)
Asian, non-Hispanic	10.7	83.9 (81.0–86.8)	13.4 (10.9–15.9)	2.8 (1.9–3.7)
Other race, non-Hispanic	4.6	71.7 (67.0–76.4)	18.0 (14.2–21.8)	10.3 (7.0–13.6)
**Insurance status, 18–64 y**				
Any private insurance	135.5	83.6 (82.8–84.4)	13.7 (12.9–14.5)	2.6 (2.3–2.9)
Public insurance only	19.4	66.9 (64.9–68.9)	21.7 (19.9–23.5)	11.4 (10.1–12.7)
Uninsured	36.7	91.9 (90.9–92.9)	6.7 (5.8–7.6)	1.5 (1.0–2.0)
**Insurance status, ≥65 y^c^**				
Medicare only	15.6	34.8 (31.8–37.8)	43.3 (40.1–46.5)	21.9 (19.3–24.5)
Medicare and private	20.3	32.4 (29.7–35.1)	43.2 (40.4–46.0)	24.4 (22.0–26.8)
Medicare and other public	4.0	25.2 (20.6–29.8)	36.8 (31.8–41.8)	38.0 (32.3–43.7)

Abbreviation: CI, confidence interval.

a Percentages may not add to 100 because of rounding.

b As defined by the US Department of Health and Human Services workgroup on multiple chronic conditions.

c The small number of people who did not fit into any of these categories were excluded.

The proportion of adults treated for MCC increased with age. In 2009, 67% of people aged 65 or older were treated for 2 or more chronic conditions and 24.6% were treated for 4 or more conditions. In contrast, 31.3% of adults aged 45 to 64 were treated for MCC and 7.0% were treated for 4 or more conditions. An estimated 5.8% of people aged 18 to 44 were treated for MCC.

Adult women were slightly more likely to be treated for MCC than adult men (27.4% vs 23.2%). Among adults aged 45 to 64, a slightly higher proportion of women than men were treated for 4 or more chronic conditions (8.1% vs 5.9%).

White non-Hispanic adults (28.5%) were most likely to be treated for MCC; Hispanic (14.4%) and Asian adults (16.2%) were least likely. Among adults aged 18 to 64, 33.1% of those with public insurance were treated for 2 or more chronic conditions compared with 16.3% of those with private insurance and 8.2% who were uninsured. Among adults aged 65 or older, 65.2% of those with only Medicare coverage and 67.6% of those with Medicare and supplemental private insurance were treated for MCC compared with 74.8% of those with Medicare and other public insurance (primarily Medicaid).

### Health care use and expenditures for MCC

The likelihood of having at least 1 hospital stay in 2009 varied from 5.3% among adults not treated for MCC to 27.7% among those treated for 4 or more chronic conditions. Comparing these same groups, the proportion with at least 1 emergency department visit varied from 11.1% to 29.7% (Table 2). Virtually all adults treated for 2 or more chronic conditions had prescribed medicine purchases and nearly all had ambulatory medical care in offices or hospital outpatient departments. In contrast, 65.5% of adults not treated for MCC had ambulatory medical visits and just over half (56.2%) had prescribed medicine purchases. Moreover, the average numbers of ambulatory visits and prescribed medicine purchases increased substantially with the number of treated chronic conditions. These patterns were similar across all age groups.

**Table 2:** Health Care Use by Age Group and Number of Treated Chronic Conditions, **a** Medical Expenditure Panel Survey, 2009

Type of Health Care	No. of Treated Chronic Conditions, Estimate (95% CI)		

	0 to 1	2 to 3	4 or more
**≥18 y**			
**Ambulatory visits **			
Percentage with ≥1 visit	65.5 (64.4–66.6)	96.0 (95.2–96.8)	98.4 (97.5–99.3)
Average no. of visits for those with ≥1 visit	6.2 (6.0–6.4)	11.4 (10.7–12.1)	17.0 (15.9–18.1)
**Emergency department visits**			
Percentage with ≥1 visit	11.1 (10.4–11.8)	17.1 (15.5–18.7)	29.7 (27.1–32.3)
Average no. of visits for those with ≥1 visit	1.3 (1.3–1.3)	1.5 (1.4–1.6)	1.6 (1.5–1.7)
**Inpatient stays **			
Percentage with ≥1 stay	5.3 (4.9–5.7)	13.8 (12.5–15.1)	27.7 (25.3–30.1)
Average no. of stays for those with ≥1 stay	1.2 (1.2–1.2)	1.4 (1.3–1.5)	1.5 (1.4–1.6)
**Prescribed medicine purchases **			
Percentage with ≥1 purchase	56.2 (55.1–57.3)	99.2 (98.9–99.5)	100.0
Average no. of purchases for those with ≥1 purchase	9.1 (8.8–9.5)	28.6 (27.7–29.5)	56.8 (54.2–59.4)
**18–44 y**			
**Ambulatory visits **			
Percentage with ≥1 visit	61.0 (59.7–62.3)	95.0 (92.5–97.5)	96.5 (89.7–103.3)
Average no. of visits for those with ≥1 visit	5.6 (5.4–5.9)	12.5 (10.8–14.2)	15.7 (11.7–19.7)
**Emergency department visits **			
Percentage with ≥1 visit	12.4 (11.5–13.3)	26.2 (21.4–31.0)	40.5 (27.9–53.1)
Average no. of visits for those with ≥1 visit	1.3 (1.3–1.3)	1.9 (1.6–2.2)	2.2 (1.7–2.7)
**Inpatient stays **			
Percentage with ≥1 stay	5.5 (5.0–6.0)	11.9 (8.7–15.1)	27.4 (14.3–40.5)
Average no. of stays for those with ≥1 stay	1.2 (1.2–1.2)	2.7 (2.4–3.0)	1.7 (1.3–2.1)
**Prescribed medicine purchases **			
Percentage with ≥1 purchase	50.7 (49.3–52.1)	98.3 (96.9–99.7)	100.0
Average no. of purchases for those with ≥1 purchase	7.3 (6.9–7.7)	27.5 (25.3–29.8)	63.9 (52.5–75.4)
**45–64 y**			
**Ambulatory visits **			
Percentage with ≥1 visit	70.7 (69.2–72.2)	95.8 (94.8–96.8)	99.2 (98.3–100.1)
Average no. of visits for those with ≥1 visit	6.7 (6.3–7.1)	10.4 (9.5–11.3)	16.9 (15.0–18.8)
**Emergency department visits**			
Percentage with ≥1 visit	8.9 (8.0–9.8)	15.3 (13.1–17.5)	32.7 (28.5–36.9)
Average no. of visits for those with ≥1 visit	1.2 (1.1–1.3)	1.4 (1.3–1.5)	1.6 (1.4–1.8)
**Inpatient stays**			
Percentage with ≥1 stay	3.7 (3.0–4.4)	11.3 (9.6–13.0)	23.6 (19.9–27.3)
Average no. of stays for those with ≥1 stay	1.2 (1.1–1.3)	1.4 (1.3–1.5)	1.6 (1.5–1.7)
**Prescribed medicine purchases **			
Percentage with ≥1 purchase	62.1 (60.5–63.7)	99.5 (99.1–99.9)	100.0
Average no. of purchases for those with ≥1 purchase	10.7 (10.2–11.3)	28.1 (26.8–29.4)	63.0 (58.8–67.2)
**≥65 y**			
**Ambulatory visits**			
Percentage with ≥1 visit	79.0 (76.1–81.9)	96.6 (95.5–97.7)	98.0 (96.6–99.4)
Average no. of visits for those with ≥1 visit	8.6 (7.8–9.4)	12.0 (10.9–13.1)	17.1 (15.7–18.6)
**Emergency department visits**			
Percentage with ≥1 visit	10.5 (8.6–12.4)	16.0 (13.9–18.1)	27.3 (23.3–31.3)
Average no. of visits for those with ≥1 visit	1.2 (1.1–1.3)	1.4 (1.3–1.5)	1.6 (1.4–1.8)
**Inpatient stays **			
Percentage with ≥1 stay	10.7 (8.6–12.8)	17.4 (15.2–19.6)	30.0 (26.5–33.5)
Average no. of stays for those with ≥1 stay	1.4 (1.3–1.5)	1.4 (1.3–1.5)	1.5 (1.4–1.6)
**Prescribed medicine purchases **			
Percentage with ≥1 purchase	74.3 (71.5–77.1)	99.3 (98.8–99.8)	100.0
Average no. of purchases for those with ≥1 purchase	12.5 (11.5–13.5)	29.5 (28.1–31.0)	52.9 (49.6–56.3)

Abbreviation: CI, confidence interval.

a As defined by the US Department of Health and Human Services workgroup on multiple chronic conditions. Estimates expressed as percentages, unless otherwise indicated.

Average expenditures for all medical care in 2009 were $8,478 among participants treated for 2 to 3 chronic conditions and $16,257 among those treated for 4 or more chronic conditions (Table 3). When restricted to treatment of chronic conditions only, these averages were $3,693 and $8,935, respectively. In contrast, the average expenditure for all conditions among adults who were not treated for MCC was $2,367.

**Table 3:** Health Care Expenditures^a^ by Age Group and Number of Treated Chronic Conditions,^b^ Medical Expenditure Panel Survey, 2009

Age, y/Expenditure	No. of Treated Chronic Conditions, Estimate (95% CI)		

	0 to 1	2 to 3	4 or more
**≥18**			
Percentage with expenditures >0	72.4 (71.4–73.4)	100.0	100.0
Average expenditures, $	2,367 (2,245–2,489)	8,478 (7,884–9,072)	16,257 (14,954–17,560)
Average expenditures for treated chronic conditions, $	408 (349–467)	3,693 (3,350–4,036)	8,935 (8,002–9,868)
**18–44**			
Percentage with expenditures >0	67.9 (66.6–69.2)	100.0	100.0
Average expenditures, $	1,862 (1,740–1,984)	8,165 (6,707–9,623)	14,746 (10,222–19,270)
Average expenditures for treated chronic conditions, $	251 (188–314)	4,004 (2,840–5,168)	8,733 (5,624–11,842)
**45–64**			
Percentage with expenditures >0	77.5 (76.1–78.9)	100.0	100.0
Average expenditures, $	2,721 (2,492–2,950)	8,129 (7,223–9,035)	17,685 (15,168–20,202)
Average expenditures for treated chronic conditions, $	462 (366–558)	3,786 (3,292–4,280)	8,914 (7,232–10,596)
≥**65**			
Percentage with expenditures >0	86.9 (84.5–89.3)	100.0	100.0
Average expenditures, $	4,878 (4,092–5,664)	8,979 (8,093–9,865)	15,553 (13,946–17,160)
Average expenditures for treated chronic conditions, $	1,420 (1,052–1,788)	3,483 (3,034–3,932)	8,961 (7,769–10,153)

Abbreviation: CI, confidence interval.

a Excludes Medical Expenditure Panel Survey expenditures not tied to specific conditions (ie, dental and other medical expenditures).

b As defined by the US Department of Health and Human Services workgroup on multiple chronic conditions.

Average medical expenses generally increased with age among those not treated for MCC. However, no significant variation was seen in either total expenses or expenses for chronic conditions between younger (18–44), middle-aged (45–64), and older adults (65 or older) with MCC.

### Treated prevalence of MCC dyads and triads

Among adults treated for MCC, hypertension and hyperlipidemia was the most common dyad combination regardless of age or sex. Among 4 groups examined by sex and age (45-64, 65 or older), the proportion treated for both conditions ranged from 42.2% for women aged 45 to 64 to 60.8% for men 65 or older. The combinations of diabetes with hypertension or hyperlipidemia were also common among all sex and age groups (ranging from 21.0% for women aged 65 or older to 28.3% for men aged 65 or older). Additional common dyads for people aged 65 or older were coronary artery disease and hyperlipidemia (32.4%) and coronary artery disease and hypertension (31.4%) for men and hypertension and arthritis for women (22.1%). Depression and hypertension or hyperlipidemia were also among the top 5 dyads for women aged 45 to 64 (19.1% and 15.6%, respectively).

Among adults treated for at least 3 chronic conditions, the combination of hypertension, hyperlipidemia, and diabetes was a common triad regardless of age or sex (ranging from 27.7% for women aged 65 or older to 37.5% for men aged 45-64). In addition, hypertension, hyperlipidemia, and coronary artery disease was the most common triad of treated conditions for men aged 65 or older (38.0%) and was prevalent among men aged 45 to 64 (24.4%) and women aged 65 or older (20.4%). Other triad combinations with prevalence of at least one-fifth of those treated for 3 or more chronic conditions were hypertension, hyperlipidemia, and cancer (22.5%) for men aged 65 or older, and hypertension, hyperlipidemia, and arthritis for women aged 65 or older (22%).

## Summary

National estimates of treated prevalence and health care expenditures associated with MCC for the civilian noninstitutionalized population can be derived from MEPS data. According to 2009 MEPS data, 31.3% of adults aged 45 to 64 and 67.1% of those aged 65 or older in the US civilian noninstitutionalized population were treated for MCC identified as prevalent and potentially amenable to public health or clinical interventions. MEPS estimates for treated prevalence and expenditures vary among population subgroups and may grow appreciably in the near future because of such factors as the aging of the population (4) and high obesity rates (5,6). This trend would exacerbate public policy concerns related to prevention, treatment, and the cost of care for people with MCC.

The data in this article show that expenditures increase substantially with number of MCC treated; in 2009, the average expense among people with 4 or more chronic conditions was almost double that for people with 2 to 3 conditions and approximately 7 times greater than for people treated for no chronic conditions or only 1 chronic condition. Consequently, reducing the number of chronic conditions among people with such conditions may generate substantial medical care savings. Another finding with potential policy implications is that no significant variation was found in medical expenditures between younger, middle-aged, and older adults with 2 or more treated chronic conditions. This lack of variation by age suggests that a strategy to reduce the prevalence of MCC among younger adults, who generally have lower health expenses and are at lower risk of chronic conditions than adults aged 65 or older, is an area that may have implications for controlling health care costs.

Although MEPS is a unique source of nationally representative information on treated prevalence and health care expenditures, the survey has strengths and limitations for examining issues related to MCC. For example, MEPS is well suited for estimating treated prevalence because conditions are ascertained in conjunction with comprehensive data collection on medical events. However, evidence suggests that MEPS-HC respondents underreport medical events (16) and are best able to accurately identify salient or broadly classified medical conditions (17). Nonetheless, in conjunction with data from other DHHS sources, MEPS data provide relevant information for policy makers seeking to address the complex issues related to treatment of people with MCC.
